# Relationship of resting echocardiography combined with serum micronutrients to the severity of low-gradient severe aortic stenosis

**DOI:** 10.1515/med-2024-1128

**Published:** 2025-11-03

**Authors:** Zhangxin Fan, Fang Lin

**Affiliations:** Department of Cardiology, Putian the 95th Hospital, No. 485, Dongyan Road, Gongchen Street, Licheng District, Putian, Fujian, 351100, China; Department of Cardiology, Putian the 95th Hospital, Putian, Fujian, 351100, China

**Keywords:** echocardiography, dobutamine stress echocardiography, serum micronutrients, aortic stenosis, low-gradient aortic stenosis

## Abstract

**Objective:**

This study investigated the relationship between serum magnesium, phosphorus, and calcium and low-gradient severe aortic stenosis (LG-AS).

**Methods:**

Dobutamine stress echocardiography (DSE) and resting echocardiography were performed on 73 patients with LG-AS. A projected aortic valve area (AVA_proj_) ≤ 1 cm^2^ or indexed AVAI_proj_ < 0.60 cm^2^/m^2^ was defined as true-severe AS. Serum magnesium, phosphorus, and calcium were compared. The correlation between serum micronutrients and resting echocardiographic parameters was analyzed. The efficacy of serum micronutrients and resting echocardiographic parameters in predicting true-severe AS in LG-AS was assessed.

**Results:**

DSE diagnosed true-severe AS in 43 patients and pseudo-severe AS in 30 patients. AVA and AVAI were smaller, and the mean pressure gradient (PG) was higher in patients with true-severe AS. Serum calcium and phosphorus were higher, and serum magnesium was lower in patients with true-severe AS. Serum calcium was positively correlated with AVA and AVAI. Serum magnesium had a moderately strong correlation with mean PG. Serum micronutrients had good predictive value for true-severe AS and AVAI, and mean PG improved the predictive value of serum micronutrients.

**Conclusion:**

Serum micronutrients have diagnostic values for true-severe AS in patients with LG-AS.

## 1 Introduction

Aortic stenosis (AS), a prevalent condition affecting the heart valve, markedly reduces life span upon developing symptoms [1,2]. Accurate grading of the severity of AS is critical in determining the appropriate treatment. Echocardiographic evaluation of patients is assessed and categorized according to the Guidelines of the European Association of Cardiovascular Imaging and the American Society of Echocardiography [3]. Severe AS is defined by small aortic valve area (AVA < 1 cm^2^ or indexed AVI [AVAI] < 0.6 cm^2^/m^2^) or high mean pressure gradient (PG > 40 mmHg, manifested by high aortic valve flow velocity). Nonetheless, the severity determined by the aortic valve PG does not consistently align with the severity outlined by AVA. Considering that the aortic valve PG depends on flow dynamics [4,5], severe AS patients with diminished left ventricular ejection fraction (LVEF) or those in a low-flow condition with maintained LVEF, might exhibit a reduced mean PG, known as low-gradient aortic stenosis (LG-AS) [6]. These patients may present with pseudo-severe AS due to incomplete opening of the stenotic valve. Patients with low-gradient AS are broadly categorized into three groups: (1) low-flow, low-gradient AS (LFLG-AS; LVEF < 50%), paradoxical low-flow, low-gradient AS (pLFLG-AS; LVEF ≥ 50% with aN indexed stroke volume, SVI, < 35 mL/m^2^), or normal flow, low-gradient AS (NFLG-AS; LVEF ≥ 50%, SVI ≥ 35 mL/m^2^) [7].

Distinguishing true-severe AS from pseudo-severe AS is vital, as individuals with true-severe AS typically gain from aortic valve replacement [8]; whereas, patients with pseudo-severe AS may not benefit from surgical intervention but require intensive pharmacologic therapy and close follow-up [9]. Dobutamine stress echocardiography (DSE) is recommended for the diagnosis of severe AS in LG-AS [10,11]. That is, the projected aortic valve area (AVAproj) at normal transvalvular flow velocity is superior to conventional resting Doppler echocardiographic parameters and can be used to differentiate between pseudo- and true-severe AS [12]. In contrast to the noninvasive nature and easy performance of resting echocardiography, DSE can cause side effects and adverse reactions in patients [13].

Traditionally, magnesium, phosphorus, and calcium are identified as micronutrients linked to maintaining bone health or managing chronic kidney disease [14] and have also been implicated in the risk of cardiovascular disease and subclinical coronary atherosclerosis. There is significant evidence that these elements also influence the occurrence and development of heart valve disease. Aortic valve stenosis, which is primarily caused by calcium deposits narrowing the valve, is the most common valvular heart condition [15]. Multifactorial calcification is caused by mechanical stress, lipid infiltration, and accumulation in inflamed tissue, ultimately causing fibrosis, thickening of the lobules, and calcification [16]. Deficiencies of magnesium may cause impaired cellular metabolic function, mitochondrial dysfunction, and endothelial dysfunction, which further harms the body [17]. Furthermore, cardiovascular diseases may be associated with abnormal phosphorus levels due to vascular calcification, inflammation, and oxidative stress [17]. Many studies have confirmed the role of these trace elements in aortic calcification. For example, elevated serum calcium and phosphorus levels promote AS through vascular calcification [18]. Decreased serum magnesium levels contribute to increased vascular calcification, which increases cardiovascular mortality [19]. Despite the association of these micronutrients with cardiovascular disease, there are limited data on the relationship between these micronutrients and the severity of AS.

This study was to assess the validity of serum magnesium, phosphorus, and calcium levels versus resting echocardiographic parameters in predicting DSE outcomes in patients with LG-AS.

## 2 Materials and methods

### 2.1 Patients

This was a cross-sectional cohort study. Patient data were collected at Putian the 95th Hospital. Seventy-three patients (age 66.9 ± 13.8 years, 41 males and 32 females) who underwent DES in Putian the 95th Hospital after echocardiographic diagnosis of LG-AS over a 2-year period (2022.03-2024.03) were studied. LG-AS was defined as an echocardiographic demonstration of a mean PG < 40 mmHg, SVI < 35 mL/m^2^, AVAI < 0.6 cm^2^/m^2^, or AVA < 1 cm^2^ (3).

The following patients were excluded: (1) atrial fibrillation or atrial tachycardia, (2) ventricular pacing rhythm, (3) mitral stenosis of more than mild severity, (4) moderate or greater aortic, mitral, or tricuspid regurgitation, (5) acute pulmonary edema, and (6) end-stage renal disease.

All patients were diagnosed with LG-AS by initial echocardiography and subsequently diagnosed with true-severe AS (total 43, age 65.2 ± 15.3 years, 23 males and 20 females) and pseudo-severe AS (total 30, age 66.4 ± 12.3 years, 18 males and 12 females) by DSE. This study was approved by the Putian the 95th Hospital Institutional Review Board, and consent was obtained from the patients or their families.

### 2.2 Collection of general patient information

General information about the patients was collected, including gender, age, height, and weight, and the body surface area was calculated [20]. Information was obtained about patients’ combined diabetes, hypertension, dyslipidemia, coronary artery disease, chronic kidney disease, and use of drugs (corticosteroids, gastric medicine, parathyroid hormone, diuretics, antibiotics, and warfarin) or supplements (gluconate oral solution, vitamin D containing tablets or capsules, sodium glycerophosphate injection, potassium magnesium aspartate tablets, magnesium sulfate injection, etc.) that affect calcium, magnesium, and phosphorus.

### 2.3 Echocardiography

All echocardiograms were performed following the focused updated current guidelines of the European Association of Cardiovascular Imaging and the American Society of Echocardiography (3). The instruments used were Philips IE33 or Epiq 7C (Philips Healthcare, Andover, MA). All echocardiograms were reviewed by a board-certified echocardiography investigator with no knowledge of all other imaging and clinical findings. The left ventricular diastolic diameter, systolic diameter, and stroke volume were assessed. The data on Doppler flow were collected in pulsed-wave mode from the LV outflow tract (LVOT) area and in continuous-wave mode from the aortic valve, utilizing transducers to achieve peak velocity. The mean PG was calculated using the streamlined Bernoulli’s principle. The left ventricular ejection fraction (LVEF) was measured using the biplane Simpson method. The LVOT diameter was measured in the parasternal long-axis view, and the measurement was used to obtain pulsed-wave Doppler data. The mean transvalvular flow rate (*Q*), calculated by dividing the stroke volume by the LVEF time, was obtained using the continuity equation to calculate AVA (cm^2^). AVAI is expressed by dividing by the body surface area (cm^2^/m^2^); AVAI < 0.60 was considered severe. For these parameters, measurements from at least three cycles were averaged.

### 2.4 DSE

DSE was performed by a specialized sonographer in the echocardiography laboratory. Initially, dobutamine was administered at 5 μg/kg/min, increasing to a peak of 20 μg/kg/min every 5 min. At every phase of DSE, measurements of AVA and gradients were taken. The diameter of LVOT was gauged solely in a resting state and was deemed steady throughout the DSE process. Peak stress measurements were recorded at the peak of the mean PG during DSE, which might not align with the last stage of the highest dose of dobutamine. AVAproj at a standard flow rate of 250 mL/s was then calculated. AVAproj was exponentiated by the body surface area. AVA_proj_ ≤ 1 cm^2^ or an exponentiated AVAI_proj_ < 0.60 cm^2^/m^2^ was defined as true severe AS. The AVA_proj_ and AVAI_proj_ equations are as follows (10):

\[{{\mathrm{AVA}}}_{{\mathrm{proj}}}=\frac{({{\mathrm{AVA}}}_{{\mathrm{peak}}}-{{\mathrm{AVA}}}_{{\mathrm{rest}}})}{{Q}_{{\mathrm{peak}}}-{Q}_{{\mathrm{rest}}}}\times (250-{Q}_{{\mathrm{rest}}})+{{\mathrm{AVA}}}_{{\mathrm{rest}}},]\]

\[{{\mathrm{AVAI}}}_{{\mathrm{proj}}}=\frac{{{\mathrm{AVA}}}_{{\mathrm{proj}}}}{{\mathrm{Body\; surface\; area}}},]\]

where AVA_rest_ and *Q*_rest_ are AVA and *Q* at rest, and AVA_peak_ and *Q*_peak_ are AVA and *Q* measured by peak load echocardiography.

### 2.5 Serum micronutrient measurements

Venipuncture was performed early after fasting for at least 12 h. The serum was separated by centrifugation (3,000 rpm for 15 min) at 4°C, all within a 90-min timeframe. For standard testing, samples were dispatched to the lab, while the rest were preserved at −70°C. The levels of calcium, phosphorus, and magnesium in serum were measured employing the Arsenazo III technique (NIPRO, Osaka, Japan), the dicarbonyl blue-I method, and the molybdate blue colorimetric technique (Sekisui Medical, Tokyo, Japan), respectively. Variation coefficients of inter and intra-assays for serum micronutrient concentrations remained below 2.4%. The bromocresol green assay (Wako Pure Chemical Industries, Osaka, Japan) was employed to gauge serum albumin concentrations. Variability coefficients for serum albumin levels, both inter- and intra-assays, remained below 2.8%. Serum calcium was adjusted for serum albumin if serum albumin was less than 4.0 mg/dL using the following formula: Corrected calcium = measured total calcium (mg/dL) + 0.8 × (4.0 serum albumin (g/dL) [21]. All reported results for calcium were based on serum albumin-corrected variables.

### 2.6 Data statistics

Statistical analyses were performed using SPSS 22.0 software (SPSS Inc., Chicago, IL, USA). The Shapiro–Wilk test was used to determine the normality of the data. Measurement data are shown as mean ± standard deviation (X̅ ± S). *t*-test (two groups) was used for comparison between two groups for data on continuous variables in a normal distribution; the Mann–Whitney U test was used for comparison between two groups for data on continuous variables in a skewed distribution. Count data are expressed as frequencies (*N*) and ratios (%), and Chi-square or Fisher’s exact tests were used. Spearman’s test was used to analyze the correlation between serum micronutrients and echocardiograms, and Bonferroni correction was used for multiple comparisons. The sample size of the study was estimated using G*Power software version 3.1.9.2 (Kiel, Germany) with a significance level of *α* = 0.05, a power of 1 – *β* = 0.8, and an effect size of *d* = 0.5, and two-sided tests were performed. *P* < 0.05 was considered statistically significant. Data were plotted using GraphPad Prism 8 (GraphPad, San Diego, CA, USA). Predictive values of serum micronutrient combinations were obtained using binary logistic analysis. Receiver operating characteristic curves (ROCs) and calculation of the area under the ROC curve (AUC) metrics were used to assess the value of true AS in patients with LG-AS. MedCalc software was used to compare whether there was a statistically significant difference in the AUCs between metrics. A *P* value of <0.05 was considered statistically significant. AUCs were compared with the AUCs of the different metrics. A *P* value of <0.05 was considered statistically significant.


**Informed consent:** Written informed consent was provided by all patients prior to the start of the study.
**Ethics statement:** The present study was approved by the Ethics Committee of Putian the 95th Hospital (IRB20210302). All procedures were performed in accordance with the ethical standards of the Institutional Review Board and the Declaration of Helsinki, and its later amendments or comparable ethical standards.

## 3 Results

### 3.1 Echocardiography of the patients

The mean age of 73 patients was 66.9 ± 13.8 years; 41 were males and 32 were females, of which 3 (4.11%) patients had bicuspid aortic valve. Forty-three (58.90%) of the 73 patients were diagnosed with true-severe AS by DSE, and the remaining 30 (41.10%) were diagnosed with pseudo-severe AS by DSE. Table 1 lists patient characteristics and resting echocardiographic and DSE results in patients with true-severe AS and pseudo-severe AS. Among the resting echocardiographic parameters, AVA and AVAI were smaller in patients with true-severe AS than in those with pseudo-severe AS (0.71 ± 0.19 cm^2^ vs 0.78 ± 0.16 cm^2^, *P* < 0.0001; 0.458 ± 0.084 cm^2^/m^2^ vs 0.504 ± 0.074, *P* < 0.0001). Mean PG was higher in patients with true-severe AS than in patients with pseudo-severe AS (24 ± 7 mmHg vs 21 ± 6 mmHg, *P* = 0.018). After assessment using DSE, the SVI, flow rate, AVA, AVAI, and mean PG were higher in both groups observed under DSE compared to resting echocardiography (all *P* < 0.05). There was also a statistically significant difference in the AVA, AVAI, and mean PG observed under DSE, shown to be AVA, and AVAI were smaller in true-severe AS patients than in patients with pseudo-severe AS (0.76 ± 0.21 cm^2^ vs 1.04 ± 0.18 cm^2^, *P* < 0.0001; 0.481 ± 0.061 cm^2^/m^2^ vs 0.689 ± 0.064, *P* < 0.0001). Mean PG was higher in patients with true-severe AS than in patients with pseudo-severe AS (43 ± 8 mmHg vs 32 ± 9 mmHg, *P* = 0.018). No statistically significant differences in age, gender, body surface area, and comorbid underlying diseases were found between the two groups of patients (all *P* > 0.05).

**Table 1:** Patient characteristics and resting echocardiography

	True-severe AS (*n* = 43)	Pseudo-severe AS (*n* = 30)	*P*
Age, years	65.2 ± 15.3	66.4 ± 12.3	0.746
Gender (male/female)	23/20 (53.50/46.50)	18/12 (60.00/40.00)	0.581
Body surface area (m^2^)	1.56 ± 0.32	1.54 ± 0.29	0.826
**Contraindication diseases**			
Hypertension	29 (67.44)	19 (63.3)	0.716
Diabetes	15 (34.89)	8 (26.67)	0.457
Dyslipidemia	16 (37.21)	6 (20.00)	0.115
Coronary artery disease	17 (39.53)	15 (50.00)	0.375
Chronic kidney disease	7 (16.28)	3 (10.00)	0.443
**Medications and supplements**			
Calcium	1 (2.35)	2 (6.66)	0.564
Magnesium	0 (0.00)	0 (0.00)	1.00
Phosphorus	0 (0.00)	0 (0.00)	1.00
Indexed LV mass (g/m^2^)	156 ± 40	159 ± 54	0.365
Aortic valve morphology (bicuspid/tricuspid)	16/27 (37.21)	7/23 (23.33/76.67)	0.209
LV diastolic dimension (mm)	48 ± 9	49 ± 11	0.748
LV systolic dimension (mm)	37 ± 11	37 ± 10	0.925
LVEF (%)	50 ± 14	51 ± 15	0.785
Heart rate (bpm)	73 ± 8	74 ± 10	0.426
SVI_rest_ (mL/m^2^)	36 ± 7	34 ± 9	0.657
Flow rate_rest_ (mL/m^2^)	168 ± 41	162 ± 43	0.642
AVA_rest_, cm^2^	0.71 ± 0.19	0.78 ± 0.16	0.000
AVAI_rest_ (cm^2^/m^2^)	0.458 ± 0.084	0.504 ± 0.074	0.000
Mean PG_rest_ (mmHg)	24 ± 7	21 ± 6	0.018
SVI_peak_ (mL/m^2^)	45 ± 9^△^	43 ± 9^△^	0.385
Flow rate_peak_ (mL/m^2^)	251 ± 67^△^	253 ± 71^△^	0.746
AVA_peak_, cm^2^	0.76 ± 0.21^△^	1.04 ± 0.18^△^	0.000
AVAI_peak_ (cm^2^/m^2^)	0.481 ± 0.061^△^	0.689 ± 0.064^△^	0.000
Mean PG_peak_ (mmHg)	43 ± 8^△^	32 ± 9^△^	0.000

LV: left ventricle; LVEF: left ventricular ejection fraction; SVI: stroke volume index; LVET: left ventricular ejection time; AVA: aortic valve area; AVAI: indexed aortic valve area; PG: pressure gradient; and AS: aortic stenosis. *P* < 0.05 was statistically significant. △*P* < 0.05 compared with resting echocardiography.

### 3.2 Serum micronutrients in the two groups of patients

**Figure 1:**
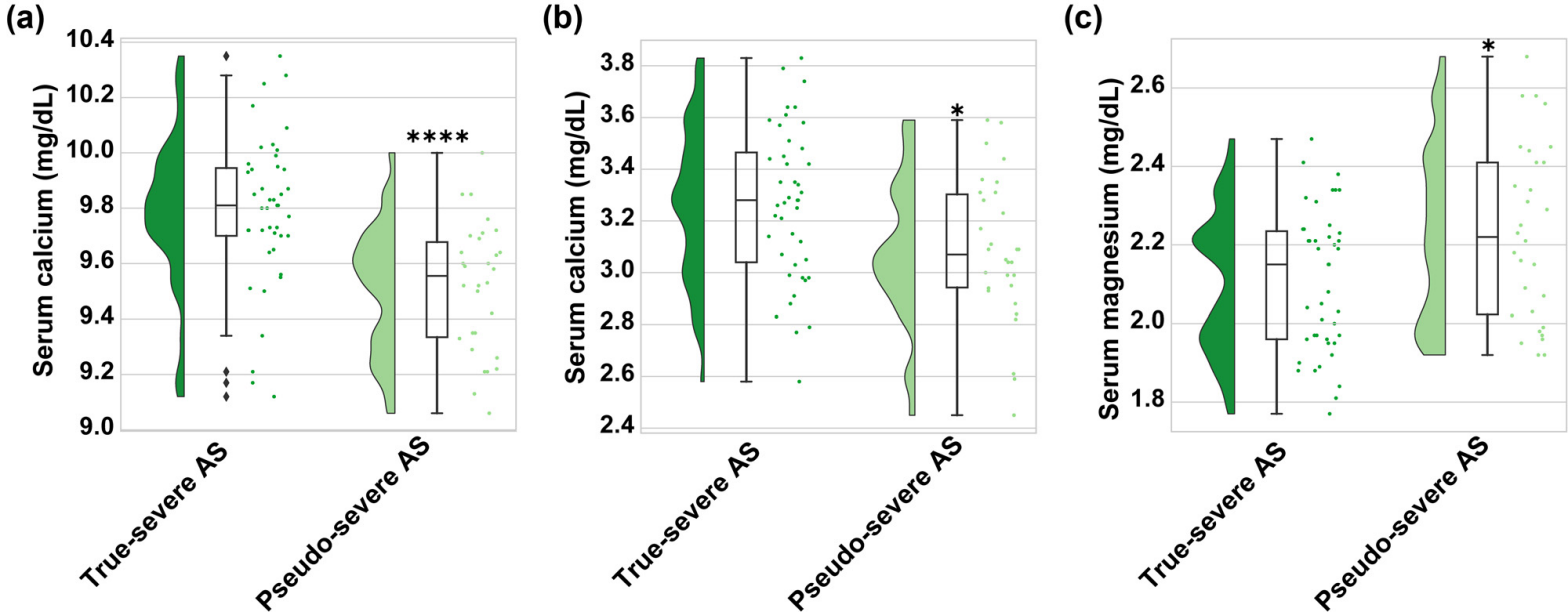
Comparison of serum micronutrients in true-severe AS and pseudo-severe AS. Levels of (a) serum calcium, (b) serum phosphorus, and (c) serum magnesium. *Student’s *t*-test and #Non-parametric test. *P < 0.05,***P < 0.001, and #*P* < 0.05. *P* < 0.05 is statistically significant.

Data analysis (Table 1) indicates that patients did not take drugs containing phosphorus or magnesium prior to measuring trace elements. A small number of patients took calcium supplements, but there is no statistical significance (*P* = 0.564). Next, serum calcium, phosphorus, and magnesium levels were measured in these patients, as shown in Figure 1. It was observed that serum calcium and phosphorus levels were higher in patients with true-severe AS than in patients with pseudo-severe AS (9.79 ± 0.27 mg/dL vs 9.52 ± 0.23 mg/dL, *P* < 0.001, Figure 1a; 3.25 ± 0.30 mg/dL vs 3.09 ± 0.27 mg/dL, *P* < 0.05, Figure 1b). Serum magnesium levels in true-severe AS patients were lower than those in pseudo-severe AS patients (2.10 ± 0.18 mg/dL vs 2.23 ± 0.22 mg/dL, *P* < 0.05, Figure 1c). Subgroups were based on median values of serum calcium 9.71 mg/dL, phosphorus 3.21 mg/dL, and magnesium 2.18 mg/dL. Patients with LG-AS, who were ultimately diagnosed with true-severe AS, were used as a risk term. They were categorized into three main groups: low risk, medium risk, and high risk. As shown in Table 2, in 73 patients with LG-AS, the percentages of those who were assessed as low, medium, and high risk based on serum micronutrients were 47.95, 30.13, and 21.92%, respectively. Among the 43 patients with true-severe AS, 14 patients (14/43, 32.56%) were at high risk, which accounted for 87.50% (14/16) of the total number of high-risk individuals, which was significantly higher than that of patients with pseudo-severe AS (2/30, 4.65%) (*P* = 0.009). There was a statistical difference in the number of patients with true- and pseudo-severe AS assessed as low risk, with the number of low-risk individuals being proportionally higher in patients with pseudo-severe AS (14/43, 9.30% vs 21/30, 48.84, *P* < 0.001). In particular, this result seems to be mainly attributable to L0 and L3, i.e., all serum micronutrients above the median value (with magnesium ≤ median) and only magnesium ≤ median (L3), which were more prevalent in patients with pseudo-severe AS.

**Table 2:** Proportion of patients with true-severe AS and pseudo-severe AS in subgroups

	True-severe AS	Pseudo-severe AS	*P*
**Low risk**			
L0	5 (11.63)	10 (23.23)	0.024
L1	4 (9.30)	0 (0.00)	0.139
L2	1 (2.33)	4 (9.30)	0.152
L3	4 (9.30)	7 (16.28)	0.182
ALL	14 (32.56)	21 (48.84)	0.000
**Medium risk**			
M1	10 (23.26)	2 (4.65)	0.106
M2	3 (6.98)	2 (4.65)	1.000
M3	2 (4.65)	3 (6.98)	0.396
ALL	15 (34.88)	7 (16.28)	0.290
**High risk**			
H	14 (32.56)	2 (4.65)	0.009
ALL	14 (32.56)	2 (4.65)	/

Data are expressed as *N* (%). Grouped according to median serum micronutrient values, low risk (risk of True-severe AS): only serum calcium ≥ median value (L1), only serum phosphorus ≥ median value (L2), or only magnesium ≤ median value (L3), where serum calcium and phosphorus are both < median value and magnesium > median value, were also included in the low-risk group (L0); medium risk: serum calcium and phosphorus both ≥ median value (M1), or magnesium ≤ median value with serum calcium ≥ median value (M2), and magnesium ≤ median value with phosphorus ≥ median value (M3); and high risk: serum calcium and phosphorus both ≥ median value, and magnesium ≤ median value.

### 3.3 Correlation analysis of serum micronutrients with resting echocardiography

**Figure 2:**
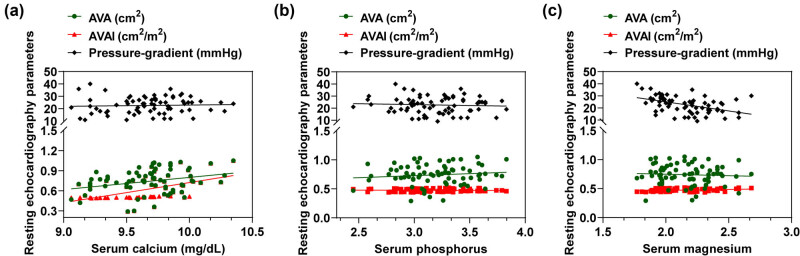
Correlation analysis plots of serum micronutrients with resting echocardiographic parameters. Correlation analysis plots of (a) serum calcium, (b) serum phosphorus, and (c) serum magnesium with resting echocardiographic parameters, including AVA, AVAI, and mean PG, respectively.

As shown in Figure 2 and illustrated in Table 3, serum calcium had a positive correlation with AVA and AVAI and a strong correlation with AVAI (rs = 0.612, *P* < 0.001) (Figure 2a). Serum magnesium had a moderately strong correlation with mean PG (rs = −0.489, *P* < 0.001) (Figure 2b). No significant correlation was observed between serum phosphorus and the above parameters (Figure 2c).

**Table 3:** Correlation coefficients between serum micronutrients and resting echocardiographic parameters

	Serum calcium (mg/dL)	Serum phosphorus (mg/dL)	Serum magnesium (mg/dL)
AVA (cm^2^)	0.249*	0.086	−0.058
AVAI (cm^2^/m^2^)	0.612***	0.102	0.201
Mean PG (mmHg)	0.106	−0.008	−0.489***

Spearman’s correlation coefficient rs was used to show correlation, 0.1–0.3 (weak correlation), 0.3–0.6 (medium strength correlation), and >0.6 strong correlation. All *P*-values are shown as corrected *P*-values (FDR program for correction). *P < 0.05 and ***P < 0.001. *P* < 0.05 is statistically significant.

### 3.4 Validity of serum micronutrients and resting echocardiographic parameters for assessment of true-severe AS

**Figure 3:**
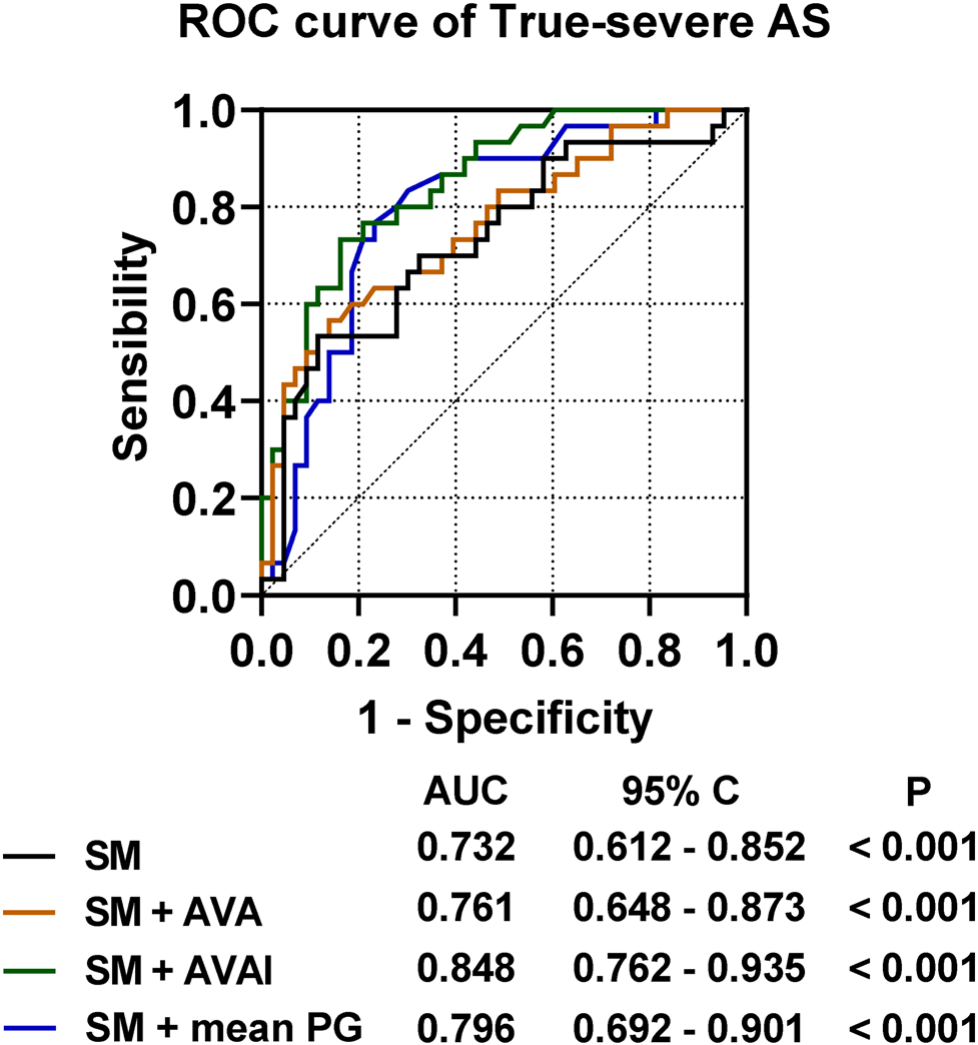
ROC curve of serum micronutrients versus resting echocardiographic parameters for predicting true-severe AS.

Next, the predictive values of the three serum micronutrients (calcium, phosphorus, and magnesium) were utilized for ROC curve analysis of true-severe AS. As shown in Figure 3, the combined predictive AUC of the three serum micronutrients was 0.732, 95% CI 0.612–0.852, which had predictive value for true-severe AS in patients with LG-AS (*P* < 0.001). AVAI combined with serum micronutrients improved the predictive value of serum micronutrients (*P* = 0.001). Mean PG combined with serum micronutrients also slightly improved the predictive value of serum micronutrients (*P* = 0.025). AVA combined with serum micronutrients had statistically significant predictive value; however, it did not improve the predictive value of serum micronutrients (*P* = 0.385).

## 4 Discussion

This study analyzed the role of resting echocardiography and serum micronutrients in predicting DSE outcomes in patients with LG-AS. It was found that (1) AVA and AVAI assessed using resting echocardiography were higher, and mean PG was lower in patients with true-severe AS; (2) serum calcium and phosphorus were higher, and serum magnesium was lower in patients with true-severe AS; and (3) using patients with true-severe AS diagnosed by DSE as a risk term and median serum micronutrient values to classify the risk group, the total number of high-risk was 16, with 87.5% (14/16) in the true-severe AS group; and (4) serum micronutrients had good predictive value for true-severe AS in patients with LG-AS, and the resting echocardiographic parameters AVAI and mean PG improved the predictive value of serum micronutrients.

Resting AVA < 1.0 cm^2^ or AVAI < 0.6 cm^2^/m^2^ was used to determine small AVA. Peak aortic valve velocity > 4 m/s and mean PG > 40 mmHg were used to determine high aortic valve velocity and high aortic valve PG, respectively [3]. However, the severity assessed by small AVA and higher mean PG is not always consistent. LG-AS is characterized by the presence of a small AVA but a lower mean PG [7]. Indeed, approximately 10–20% of severe AS have been reported as classic low-flow LG-AS with reduced LVEF, and 10–25% of severe AS with preserved LVEF have been reported as paradoxical low-flow LG-AS [6,22]. Due to its flow-dependent nature, mean PG may underestimate the severity of stenosis, whereas lower AVA or AVAI may overestimate the severity of stenosis due to incomplete opening of the valve. Small AVA due to low-flow status is referred to as pseudo-severe AS (although its actual severity is moderate) and should be differentiated from true-severe AS for the selection of appropriate therapy (8). DSE is recommended in patients with classic low-flow AS to distinguish true-severe AS from pseudo-severe AS [23,24]. A previous study also recommends DSE and AVAproj measurements in pLFLG-AS patients with preserved LVEF [25]. Critical values of AVAproj and AVAIproj are used to predict clinical outcomes between LFLG-AS and pLFLG-AS (1.0 cm^2^ and 0.6 cm^2^/m^2^, respectively) [25]. The significance of DSE seems to be the same in both patients. Nonetheless, DSE presents as a lengthy and challenging diagnostic instrument for doctors and technicians and is not recommended for clinical application. Therefore, our study focused on these two types of patients who underwent resting echocardiography and DSE to differentiate between true and pseudo-severe AS by DSE and to observe the characteristics of the differences in parameters in resting echocardiography. In addition, certain differential parameters of resting echocardiography in combination with serum indices may predict the outcome of DSE in patients with LG-AS.

Aortic calcification is one of the most important etiologic factors of AS. High serum calcium levels may lead to deposition of calcium deposition on the aortic valve, leading to sclerosis of the aortic valve, which will eventually lead to stenosis of the aortic orifice in patients [26]. In patients with end-stage renal disease, increased serum phosphorus levels also promote mitral and aortic valve calcification, whereas serum magnesium concentrations negatively correlate with vascular calcification and may be a compensatory regulatory mechanism that reduces calcium phosphate hydrolysis and vascular calcification [27]. Previous research has demonstrated that the majority of patients with severe aortic valve stenosis and a low mean transvalvular gradient, as assessed by echocardiography, exhibit significant valve calcification [28,29]. In a multiethnic group of patients with atherosclerosis, increased serum phosphorus and urinary phosphorus levels are associated with aortic valve calcification [30]. Serum magnesium is negatively associated with the prevalence of AS [15]. A previous study reports a 1.79-fold increase in the risk of AS per 0.1 mmoL/L increase in calcium concentration and a 1.47-fold increase in the risk of AS per 0.1 mmoL/L increase in phosphorus concentration [31]. In addition, another study shows a 51% increase in the risk of dying from AS for every 0.1 mmoL/L increase in serum calcium [32]. A significant correlation is known to exist between the severity of AS and the risk of death. If severe symptomatic AS is left untreated, the prognosis is poor, with a mortality rate of up to 50% at 1 year after the onset of symptoms and more than 90% at 5 years [33]. Therefore, serum micronutrients are potentially important and reliable markers of AS severity.

In the present study, serum calcium and phosphorus had higher levels, and serum magnesium had lower levels in patients with true-severe AS. This is the first study to confirm the association of serum micronutrients with AS severity. Aortic calcification represents a vascular pathology marked by the accumulation of calcium within the arterial wall. The principal pathological mechanisms contributing to the development of aortic calcification include oxidative stress, inflammation, and endothelial dysfunction. Elevated levels of blood calcium and phosphorus expedite the progression of aortic calcification by facilitating the osteogenic differentiation of vascular smooth muscle cells (VSMCs) and enhancing the expression of calcification-associated factors, such as alkaline phosphatase (ALP) and osteocalcin (OC) [34,35]. A clinical study has demonstrated that the serum calcium–phosphorus product (Ca × P) exhibits a positive correlation with the severity of aortic calcification, thereby reinforcing the critical involvement of calcium and phosphorus in vascular calcification [36]. Furthermore, magnesium ions have been shown to inhibit the proliferation and migration of VSMCs, thereby mitigating their differentiation into osteoblast-like cells. Additionally, magnesium ions can suppress inflammation and oxidative stress, consequently diminishing the damage inflicted by reactive oxygen species on the vascular wall and alleviating endothelial dysfunction [37]. It is worth noting that when endothelial dysfunction occurs, platelet activation and adhesion cannot be effectively inhibited, and thrombi are prone to form. This thrombotic state may not only directly affect the function of the aortic valve but may also indirectly aggravate the symptoms of AS by, for example, blocking blood flow [38]. In addition, in AS, the left ventricle needs to contract more strongly to pump sufficient blood, which may result in a large systolic pressure difference between the left ventricle and the aorta. This pressure difference may result in reduced blood flow to the aorta and coronary arteries, further affecting endothelial cell function and survival [39]. There may be an interaction and vicious circle between endothelial dysfunction and AS. Therefore, the present study attempted to subgroup by serum micronutrient levels. Patients with LG-AS who were eventually diagnosed with true-severe AS by DSE were used as a risk term. The results showed that a total of 16 patients were assessed as high risk, of which 14 were all present in the true-severe AS group. Subsequently, we confirmed a positive correlation of serum calcium with AVA and AVAI, with a particularly strong positive correlation with AVAI (rs = 0.612, *P* < 0.001). There was a moderately strong correlation between serum magnesium and mean PG (rs = −0.489, *P* < 0.001). However, we did not observe a significant correlation between serum phosphorus and the above parameters. Therefore, we believe that serum calcium and magnesium are potentially closely correlated with resting echocardiography. Finally, we plotted ROC curves to analyze the predictive value of true-severe AS in patients with LG-AS. As expected, the combination of the three serum micronutrients had predictive values, and the resting echocardiographic parameters AVAI and mean PG improved the predictive value of serum micronutrients.

However, this study also has limitations. First, this study is a cross-sectional cohort study that only establishes an association, not causality. Follow-up of patients over a certain time frame would provide an opportunity to identify changes in valvular gradients associated with serum nutrient elements. Second, the study cohort consisted of a relatively small number of subjects, which may limit the generalizability of the findings. A multifactorial screening for independent correlates would be more favorable to increase the reliability of the diagnostic results of the ROC curves. Finally, our results apply only to calcium, phosphorus, and magnesium.

## 5 Conclusion

Serum micronutrients are associated with the severity of AS patients. Serum micronutrients are effective in differentiating between LG-AS patients with true-severe AS, and the resting echocardiographic parameters AVAI and mean PG improve the assessment value of serum micronutrients. Based on the results of this study, it can be used as a theoretical basis for the development of predictive models for confirming the diagnosis of true-severe AS among patients with LG-AS and therapeutic strategies for slowing down the disease process in clinical treatment.
